# Deregulated Rac1 Activity in Neural Crest Controls Cell Proliferation, Migration and Differentiation During Midbrain Development

**DOI:** 10.3389/fcell.2021.704769

**Published:** 2021-09-07

**Authors:** Apurva Gahankari, Chunmin Dong, Garrett Bartoletti, Maria Galazo, Fenglei He

**Affiliations:** ^1^Department of Cell and Molecular Biology, Tulane University, New Orleans, LA, United States; ^2^Tulane Brain Institute, Tulane University, New Orleans, LA, United States

**Keywords:** Rac1, constitutive activation, neural crest, midbrain development, dopaminergic neuron, mouse

## Abstract

Mutations in *RAC1* allele are implicated in multiple brain tumors, indicating a rigorous control of Rac1 activity is required for neural tissue normal development and homeostasis. To understand how elevated Rac1 activity affects neural crest cells (NCCs) development, we have generated *Rac1^*CA*^;Wnt1-Cre2* mice, in which a constitutively active Rac1^*G*12*V*^ mutant is expressed specifically in NCCs derivatives. Our results revealed that augmented Rac1 activity leads to enlarged midbrain and altered cell density, accompanied by increased NCCs proliferation rate and misrouted cell migration. Interestingly, our experimental data also showed that elevated Rac1 activity in NCCs disrupts regionalization of dopaminergic neuron progenitors in the ventral midbrain and impairs their differentiation. These findings shed light on the mechanisms of *RAC1* mutation correlated brain tumor at the cellular and molecular level.

## Introduction

Small GTPases are essential for embryonic development and homeostasis. *Ras-related C3 botulinum toxin substrate 1* (*Rac1*), which encodes a small GTPase of the Ras superfamily, is implicated in multiple processes including actin cytoskeleton rearrangement, cell migration, and cell cycle progression (Didsbury et al., 1989; Ridley, 2001; Wittmann et al., 2003; Hall, 2005; Bosco et al., 2009; Hua et al., 2015). In cells, Rac1 protein shuttles between two states, the GTP-bound active state and GDP-bound inactive form. Formation of Rac1-GTP is catalyzed by guanine exchange factors (GEFs), which facilitate exchange of GDP for GTP (Rossman et al., 2005). On the other hand, Rac1-GTP can be inactivated by GTPase activating proteins (GAPs), which hydrolyze Rac1 bound GTP to GDP (Jaffe and Hall, 2005). GEFs and GAPs recognize and bind to specific amino acids of Rac1 and modulate its activity. Mutation in these amino acids causes deregulated Rac1 activity. For example, G12V mutation of Rac1 impairs its interaction with GAP, and causes its constitutive activation in a Rac1-GTP form (Kobayashi et al., 1998). Mutation in RAC1 and its regulators have been implicated in abnormal development and tumorigenesis (Khalil and El-Sibai, 2012). In brain tumors, multiple mutations in RAC1 putative effector domain have been identified (Hwang et al., 2004), suggesting a correlation between excessive RAC1 activity and brain tumorigenesis and abnormal development. However, the mechanisms underlying excessive Rac1 activity in regulation of neuronal tissues and brain development have not been fully elucidated.

Neural crest cells (NCCs) are a unique cell population characterized by their transient, highly migratory, and multi-potent features (Le Douarin and Kalcheim, 1999). Originated as ectodermal cells at the border of the neural plate at the dorsal neural tube, NCCs undergo epithelial to mesenchymal transition (EMT), then migrate to the ventral destinations. Lineage tracing studies demonstrate that NCCs give rise to a broad range of cell types in craniofacial and cardiovascular tissues (Chai et al., 2000; Jiang et al., 2000, 2002; Yoshida et al., 2008). NCCs derivatives are also identified in the developing midbrain (Jiang et al., 2002; Lewis et al., 2013). Neural crest development is subject to rigorous genetic control (Simões-Costa and Bronner, 2015; Bronner and Simões-Costa, 2016). While *Rac1* is required for all three germ layers formation, as *Rac1* null mutant mice die at embryonic day (E) 9.5 (Sugihara et al., 1998), tissue-specific inactivation in mice identified an important role for Rac1 in neural crest development. *Wnt1Cre* mediated *Rac1* deletion in NCCs causes embryonic lethality at E12.5 with severe midfacial clefting and cardiovascular malformation (Fuchs et al., 2009; Thomas et al., 2010). We have reported that chemical inhibition of Rac1 activity represses NCCs proliferation and migration in neural crest explant culture (He and Soriano, 2013). Together, these studies demonstrated that *Rac1* is essential for NCCs development using loss-of-function models.

At the onset of midbrain development, NCCs derived cells are detected at the midbrain-hindbrain boundary, in close proximity to the ventral midbrain dopaminergic neurons (mDA). The mDA differentiation is well documented and can be divided into three stages. First, the mDA precursors are induced within the neuroepithelium and express *Shh, Wnt1*, and other midbrain regionalization markers at E8.5–10.5. Next, these mDA progenitors undergo differentiation characterized by the expression of *neurogenin-2* (*Ngn2*) and *nuclear receptor related 1* (*Nurr1*) between E10.5 and E12.5. Finally, mDA undergo terminal differentiation and are characterized by the expression of *tyrosine hydroxylase (TH)* at E12.5 (Prakash and Wurst, 2006a,b; Gale and Li, 2008; Lewis et al., 2013). Previous studies also showed that *Rac1* is implicated in dopaminergic neuron development (Čajánek et al., 2013; Hua et al., 2015; Kim et al., 2018). However, whether augmented Rac1 activity affects mDA development has not been elucidated.

To understand how augmented Rac1 activity affects NCCs and midbrain development, we have generated *Rac1^*CA*^;Wnt1-Cre2* mice, which express Rac1^*G*12*V*^ specifically in NCCs derivatives (Srinivasan et al., 2009; Lewis et al., 2013). Our data showed that *Rac1^*CA*^;Wnt1-Cre2* can survive to adulthood with enlarged midbrain. Histological analysis revealed that cell density and organization are altered in the mutant midbrain. At the cellular level, the mutant NCCs exhibit increased proliferation rate and abnormal migration patterns. Interestingly, we have identified dramatic loss of mDA and likely impaired neuronal differentiation in the mutant ventral midbrain. These findings, together with the results from previous studies using loss-of-function models, provide a comprehensive view of *Rac1* regulation on NCCs and mDA development.

## Materials and Methods

### Animals

All animal experimentation was approved by the Institutional Animal Care and Use Committee of Tulane University. Rosa26-LSL-Rac1^*G*12*V*^ (Srinivasan et al., 2009), referred to as *Rac1*^*CA*^ and B6.Cg-*E2f1^*Tg(Wnt*1–cre)2*Sor/J*^* (Lewis et al., 2013), referred to as *Wnt1-Cre2*, are maintained on a C57BL6/J129 mixed genetic background. For embryo collection, noon of the day when vaginal plug was found in the mating female was defined as E0.5. In all the experiment, genotyped *Wnt1-Cre2* transgenic littermates were used as control.

### Histology

Staged embryos were dissected in PBS, and fixed in 4% paraformaldehyde (PFA) in PBS at 4°C. Following overnight fixation, the embryos were dehydrated by gradient ethanol washes and were embedded in paraffin. Samples were sectioned at 10 μm and subjected to standard Hematoxylin/Eosin staining and histological analysis as described (Dong et al., 2019).

### BrdU Labeling

For mouse embryos, BrdU/PBS solution was administered by IP injection into pregnant females at 5 mg/100 g body weight. 1 h later, embryos were dissected and fixed in 4% paraformaldehyde (PFA) overnight at 4°C. Samples were then dehydrated and embedded in paraffin. Immunostaining was performed on 10 μm coronal sections according to standard protocols using anti-BrdU antibody (1:400, Novus, NBP2-14890) and Alexa Fluor 594 goat anti-rabbit IgG secondary antibody (1:500, Invitrogen, A11012). Sections were counterstained with DAPI to facilitate quantification. BrdU positive cells were quantified and the data were analyzed using ImageJ (Rueden et al., 2017). For quantification, the value in *Y* axis was shown as the ration of number of positive cells against the total nuclei in the defined area. Statistical analysis was performed on data collected from three consecutive sections from each of four to five independent experiments. Statistical data are presented as mean value ± SEM and subjected to double tailed Student’s *t*-tests.

### *In situ* Hybridization and Immunohistochemistry

Section *In situ* hybridization on paraffin sections was carried out as described previously (Bartoletti et al., 2020). A 820 bp of *Ngn2* cDNA fragment was amplified using primers 5- GCCCTTCTCCACCTTCCT-3 and 5-GAAGGGCGGGACAATAGG-3, 967 bp of *Nurr1* cDNA fragment using primers 5-CGTAGCATCACCACGGACTT-3 and 5-GGCATCATCTCCTCGGACTG-3, a 520 bp of *TH* cDNA fragment using primers 5- GTCGGGTGTCTGACGATGTG-3 and 5-TGGCTCGGGTGAGTGCATA, and a 650 bp *Shh* probe (Echelard et al., 1993) were generated by PCR.

Whole-mount *in situ* hybridization was performed as previously described (He and Soriano, 2013). Briefly, E9.5 embryos were dissected in ice cold PBS and fixed in 4% PFA/PBS overnight. Following gradient washes, the embryos were dehydrated to 100% methanol, and were bleached in methanol: 30% hydrogen peroxide (4:1) solution at room temperature for 1 h. The embryos were then rinsed with PBT (PBS + 0.1% Tween-20) followed by treatment with proteinase K (10 μg/ml) in PBT at room temperature for 10 min. Subsequently, the embryos were washed with 2 mg/ml glycine in PBT, fixed with 4% PFA/PBS for 20 min, and washed in PBT twice for 5 min each time. Following wash in prehybridization buffer for 1 h at 65°C, the embryos were incubated overnight in hybridization buffer with denatured RNA probe (1 μg/mL in hybridization buffer) at 65°C. Post- hybridization washes were performed followed by incubation with 10% normal sheep serum for 1 h at room temperature, and anti-digoxigenin antibody/1% sheep serum (1:2000) at 4°C overnight. The embryos were subjected to post-antibody washes followed by color development.

For immunohistochemistry, samples were sectioned at 10 μm and subjected to standard protocols using anti-Rac1-GTP antibody (1:500, NewEast Bio, 26903), anti-cleaved caspase 3 antibody (1:500, Cell Signaling Technology, 9664), anti-TH antibody (1:1000, Millipore, AB152), and Alexa Fluor 594 goat anti-rabbit IgG secondary antibody (1:500, Invitrogen, A11012). For whole-mount immunostaining, E10.5 embryos were dissected and fixed with 4% PFA/PBS overnight. The embryos were then rinsed with PBS three times followed by wash with 0.1% Triton X-100. Following blocking in 5% normal goat serum for 1 h at room temperature, the samples were treated with anti-neurofilament-L (1:500, Cell Signaling Technology, C28E10) for 2 days at 4°C. The immunofluorescence signal was developed with Alexa Fluor 594 goat anti-rabbit IgG secondary antibody (1:500, Invitrogen, A11012). The embryos were then dehydrated with 100% MeOH and cleared with BABB solution (benzyl alcohol: benzyl benzoate at a ratio of 1:2) for imaging.

## Results

### Generation of *Rac1*^*CA*^;*Wnt1-Cre2* Mice and Validation of Rac1 Activity Augmentation in NCCs Derivatives

**FIGURE 1:**
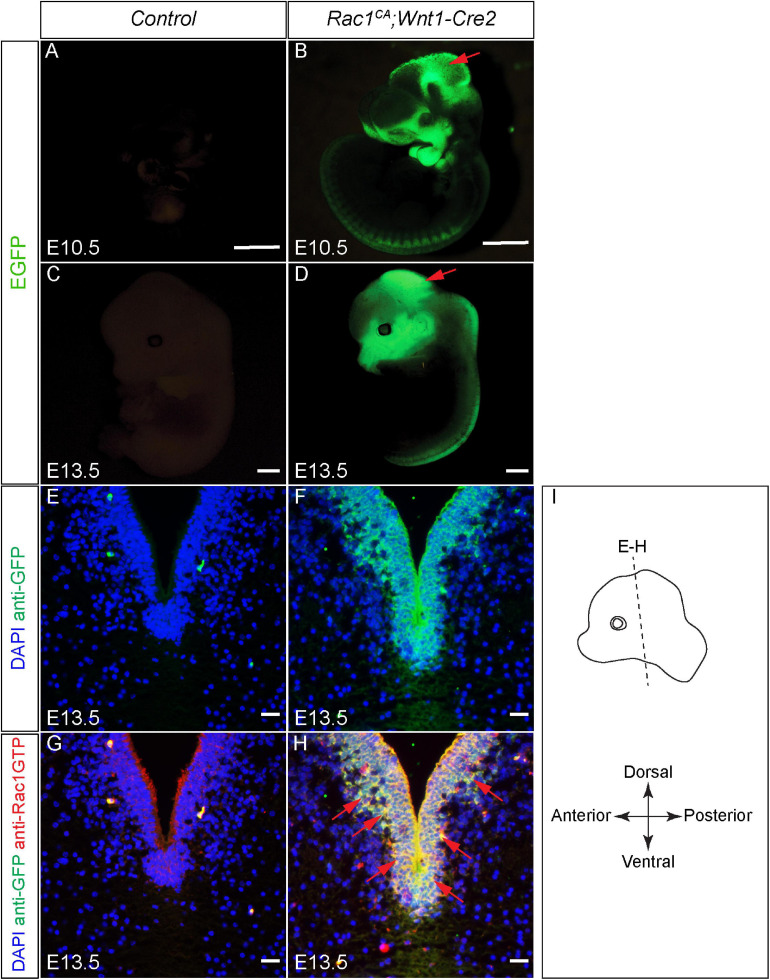
Validation of augmented Rac1 activity in *Rac1^*CA*^;Wnt1-Cre2* embryos. **(A–D)** Whole-mount view of the control **(A,C)** and *Rac1^*CA*^;Wnt1-Cre2* **(B,D)** embryo at E10.5 **(A,B)** and E13.5 **(C,D)**. **(E,F)** Immunostaining with anti-GFP antibody (green) on coronal sections of the control **(E)** and *Rac1^*CA*^;Wnt1-Cre2* **(F)** at the level of ventral midbrain at E13.5. Slides are counterstained with DAPI (blue). **(G,H)** Co-immunostaining with anti-Rac1-GTP antibody (red) and anti-GFP antibody (green) on coronal sections of E13.5 littermate control **(G)** and *Rac1^*CA*^;Wnt1-Cre2* **(H)** at the level of ventral midbrain. Slides are counterstained with DAPI (blue). Red arrows point to overlapped expression of EGFP and Rac1-GTP. **(I)** Illustration of the embryo orientation and section level in figure and following figures. Scale bar in **A–D**, 1 mm. Scale bar in **E–H**, 10 μm.

To examine the role of increased Rac1 activity in NCCs development, *Rac1*^*CA*^ has been crossed to *Wnt1-Cre2* to generate *Rac1^*CA*^;Wnt1-Cre2* mice (Srinivasan et al., 2009; Lewis et al., 2013). First we examined the expression pattern of *Rac1*^*CA*^ in the mutant. The *Rac1*^*CA*^ knock-in line was generated using *ROSA26* allele harboring sequentially a *loxP*-flanked STOP cassette, *Rac1^*G*12*V*^* cDNA and *IRES-EGFP*. When crossed to a *Cre* line and successful recombination, *Rac1^*G*12*V*^* cDNA and *IRES-EGFP* will be expressed coincidentally in cells expressing *Cre* (Srinivasan et al., 2009). In *Rac1^*CA*^;Wnt1-Cre2* mice, we have identified *EGFP* expression in *Rac1^*CA*^;Wnt1-Cre2* embryos in the craniofacial mesenchyme, midbrain, and dorsal neural tube at both E10.5 and E13.5 (Figures 1A–F, *n* = 5 at each stage), in a pattern recapitulated by *Wnt1-Cre2* triggered reporter expression in NCC derived cells (Lewis et al., 2013). Rac1-GTP overexpression is further validated by immunostaining in the sections across the midbrain of E13.5 *Rac1^*CA*^;Wnt1-Cre2* embryo (Figures 1G,H) at level indicated in Figure 1I. These results demonstrated successful expression of Rac1^*CA*^ in NCCs derived cells in the mutant mice.

### Histological Analysis of *Rac1^*CA*^;Wnt1-Cre2*

**FIGURE 2:**
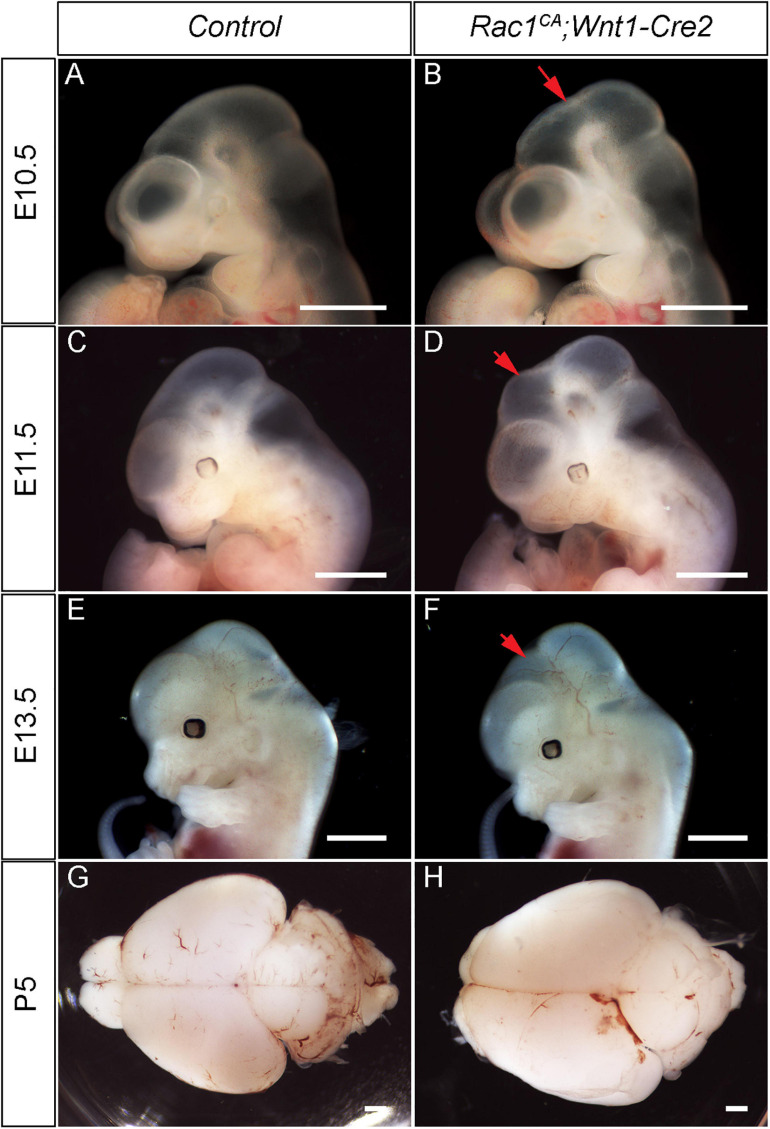
Dysregulated Rac1 activity leads to abnormal neural crest development and midbrain malformation. **(A–F)** Lateral view of the control **(A,C,E)** and *Rac1^*CA*^;Wnt1-Cre2* **(B,D,F)** embryos at E10.5 **(A,B)**, E11.5 **(C,D)**, and E13.5 **(E,F)**. Red arrows in **B,D,F** point to the midbrain of the mutant. **(G,H)** Whole-mount view of P5 brain of the littermate control **(G)** and *Rac1^*CA*^;Wnt1-Cre2* **(H)**. Scale bar in **A–H**, 1 mm.

**FIGURE 3:**
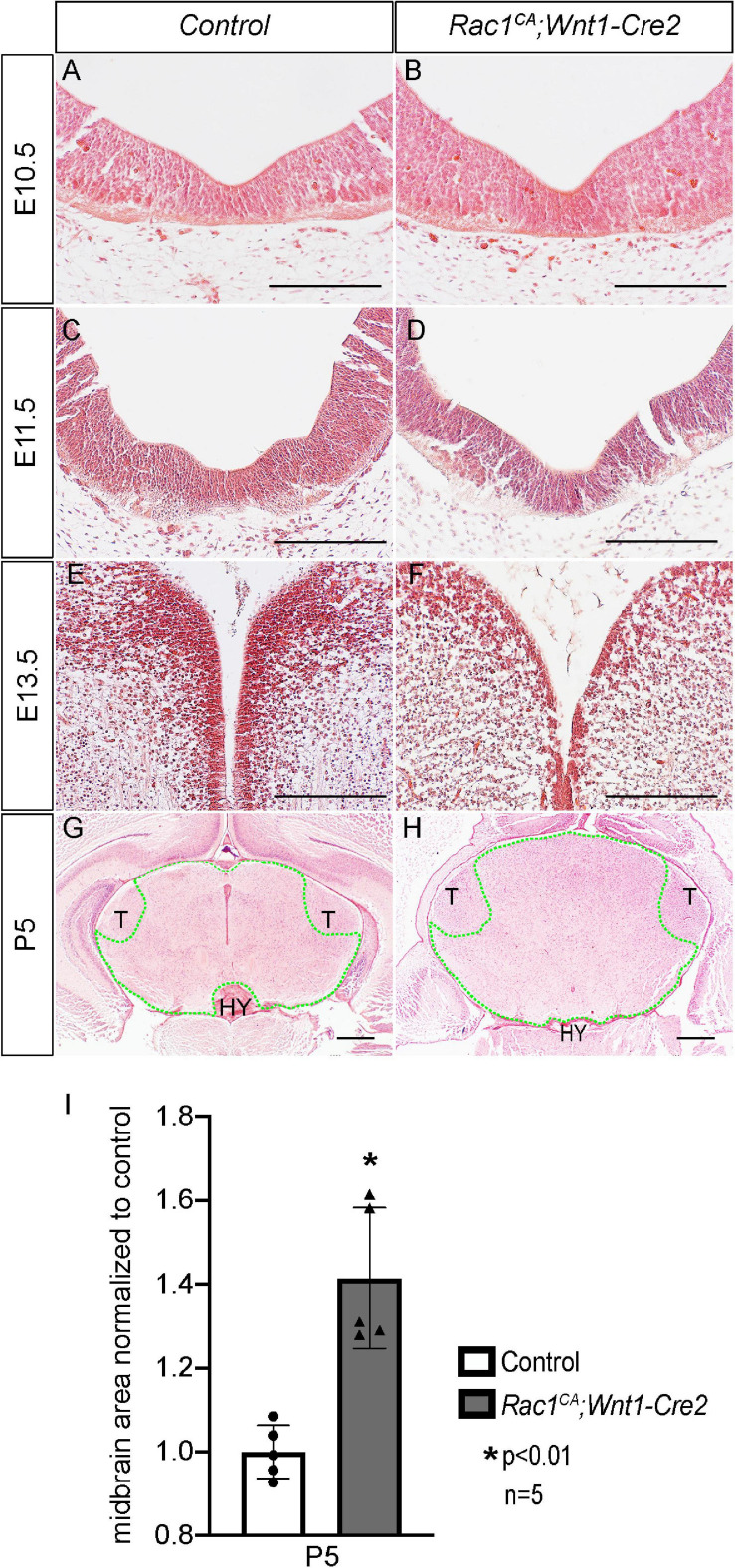
Histological analysis of *Rac1^*CA*^;Wnt1-Cre2* embryos. **(A–H)** H.E. staining on coronal sections of littermate control **(A,C,E,G)** and *Rac1^*CA*^;Wnt1-Cre2* **(B,D,F,H)** at E10.5 **(A,B)**, E11.5 **(C,D)**, E13.5 **(E,F)**, and P5 **(G,H)** at midbrain level. **(I)** Scatter plot of statistical analysis of midbrain area (outlined by green dots) quantified in **(G,H)**. Scale bar in **A–H**, 200 μm. HY, hypothalamus; T, thalamus.

**FIGURE 4:**
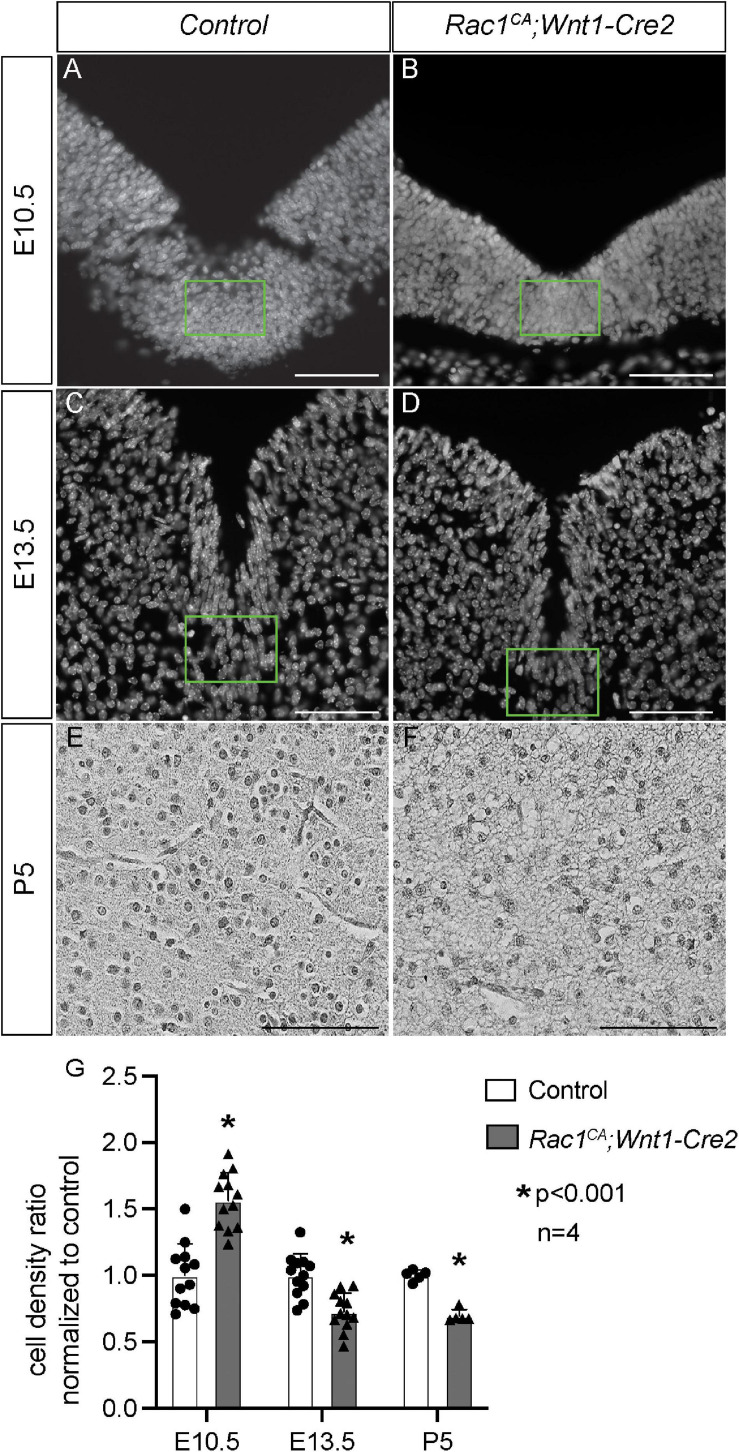
*Rac1^*CA*^;Wnt1-Cre2* mice exhibit altered cell density in ventral midbrain. **(A–C)** Grayscale conversion of DAPI staining on coronal sections of littermate control midbrain **(A,C)** and that of *Rac1^*CA*^;Wnt1-Cre2* **(B,D)** at E10.5 **(A,B)** and E13.5 **(C,D)**. Ventral midbrain tissue defined in green rectangles are used for quantification and statistical analysis. **(E,F)** Grayscale conversion of H.E. staining on coronal sections of littermate control midbrain **(E)** and that of *Rac1^*CA*^;Wnt1-Cre2* **(F)** at P5. **(G)** Quantification and statistical analysis of cell density in **A–F**. Scale bar in **A–D**, 50 μm. Scale bar in **E,F**, 200 μm.

Next, we analyzed the phenotype of the *Rac1^*CA*^;Wnt1-Cre2* mice. We found that two out of six mutant mice survived beyond P20, and both showed an enlarged head. To examine the midbrain phenotype, we have collected and analyzed mutant embryos at stages from E9.5 through P5. The mutant embryos are comparable to the control littermates at E9.5 (data not shown) and E10.5 (Figures 2A,B, *n* = 5). Morphological difference was first observed at E11.5, and the mutant embryo exhibit a “bulge” at the junction of forebrain and midbrain (Figures 2C,D, *n* = 4). This phenotype is observed throughout E13.5 (Figures 2E,F, *n* = 5) and later stages. At P5, the whole brain tissue of the mutants is enlarged compared to the controls (Figures 2G,H, *n* = 7). Hematoxylin and Eosin (H.E.) staining on cross sections at the level of midbrain confirmed that the mutant midbrain is enlarged at above stages (Figures 3A–H). Statistical analysis revealed that at P5, the mutant midbrain area is 1.42 ± 0.08 fold of the control (Figure 3I, *p* < 0.01, *n* = 5). Such a change in brain size indicates alteration of cell density or cell numbers, or a combination of both. To answer this question, we have quantified the cell density of the mutant midbrain and the littermate control. Statistical analysis results showed that the cell density of the mutant midbrain increased to 1.56 ± 0.09 fold (*p* < 0.01) of the control at E10.5 (*n* = 4), to 0.73 ± 0.06 fold (*p* < 0.01) of the control at E13.5 (*n* = 4) and decreased to 0.69 ± 0.03 fold of the control at P5 (*n* = 5) (Figures 4A–G). These results suggest that expression of Rac1^*G*12*V*^ in neural crest might altered cell proliferation rate and survival in the mutant.

### Analysis of Cell Proliferation Rate and Apoptosis in *Rac1^*CA*^;Wnt1-Cre2* Midbrain

**FIGURE 5:**
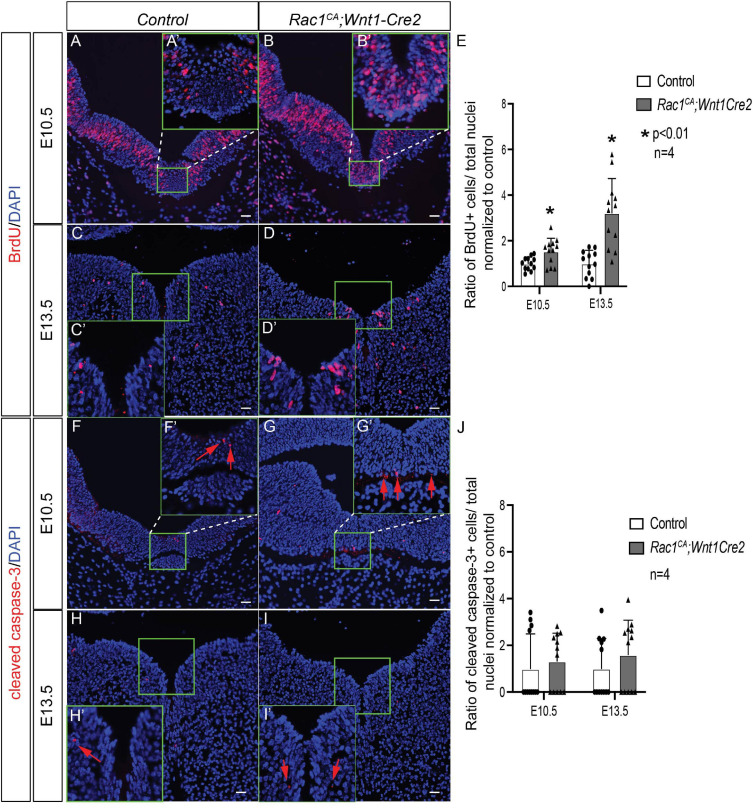
Analysis of cell proliferation and apoptosis of *Rac1^*CA*^;Wnt1-Cre2.* **(A–D)** BrdU labeling (red) on coronal sections of midbrain in littermate control **(A,C)** and *Rac1^*CA*^;Wnt1-Cre2* **(B,D)** at E10.5 **(A,B)** and at E13.5 **(C,D)**. Insets **A’–D’** are magnification of defined areas in **A–D**. **(E)** Statistical analysis of quantification results in **A–D**. **(F–I)** Immunostaining using anti-cleaved caspase 3 antibody (red) on coronal sections of midbrain in littermate control **(F,H)** and *Rac1^*CA*^;Wnt1-Cre2* **(G,I)** at E10.5 **(F,G)** and at E13.5 **(H,I)**. Insets **F’–I’** are magnification of defined areas in **F–I**. Red arrows point to cells expressing cleaved caspase 3. **(J)** Statistical analysis of quantification results in **F–I**. Cells in ventral midbrain (green rectangles in **A–D,F–I**) are quantified and used for statistical analysis. Scale bar in **A–I**, 10 μm.

To examine the role of augmented Rac1 activity in midbrain development at the cellular level, we have assayed cell proliferation rate using BrdU labeling and apoptosis using immunostaining with anti-cleaved caspase 3 antibody, respectively. Statistical analysis revealed a significant increased cell proliferation rate in mutant midbrain cells at E10.5 (Figures 5A,A’,B,B’,E; 1.54 ± 0.18 fold, *p* < 0.01, *n* = 4) and at E13.5 (Figures 5C,C’,D,D’,E; 3.13 ± 0.42 fold, *p* < 0.01, *n* = 4). On the other hand, we did not observe significant change in the ratio of apoptotic cells in the mutant compared to that in the control (Figures 5F–J) at either E10.5 (1.32 ± 0.55 fold, *p* > 0.05, *n* = 4) or at E13.5 (1.59 ± 0.56 fold, *p* > 0.05, *n* = 4). These data demonstrated that Rac1 constitutive activation promotes cell proliferation in the NCCs derived midbrain cells but does not affect apoptosis.

### Augmented Rac1 Activity Alters NCCs Migration *in vivo*

**FIGURE 6:**
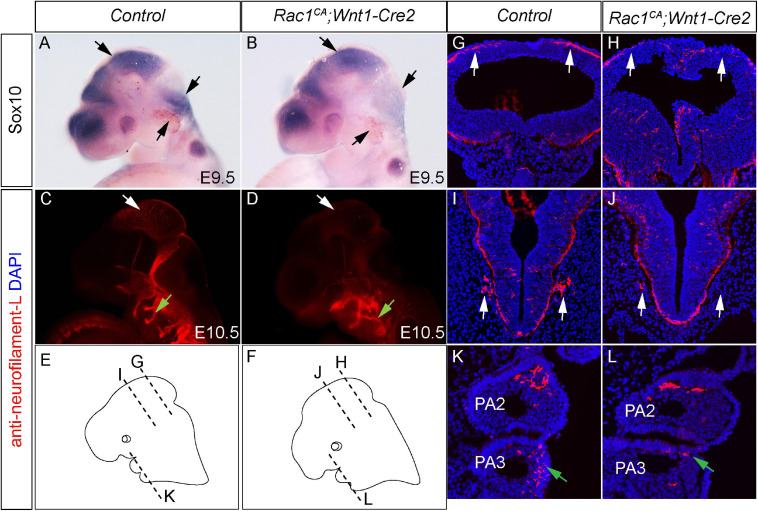
Augmented Rac1 activity alters neural crest cell migration *in vivo*. **(A,B)** Whole-mount *in situ* hybridization with Sox10 antisense mRNA probe in E10.5 littermate control **(A)** and *Rac1^*CA*^;Wnt1-Cre2* **(B)** embryos. Arrows point to counterparts in both embryos, where *Sox10* expression is altered in the mutant. **(C,D)** Whole-mount immunostaining using anti-neurofilament L antibody of E10.5 littermate control **(C)** and *Rac1^*CA*^;Wnt1-Cre2* **(D)** embryos. Blank arrows point to midbrain. Green arrows point to neurofilament in pharyngeal arch (PA) 2. **(E,F)** Diagram shows section levels in **C,D**, as illustrated in **G–L**. **(G–L)** Coronal sections of **C,D** at comparable levels illustrated in **E,F**. Blank arrows in **H,J** point to reduced neurofilament L expression in the mutant compared to that in **G,I**, respectively. Green arrow in **L** points to misguided NCCs expressing neurofilament in mutant PA3 when compared to the counterpart tissues in the control PA3 shown in **K**.

Rac1 is an essential regulator of cell migration. Previous studies have shown that *Rac1* deficiency impairs normal NCCs migration (Fort et al., 2011; He and Soriano, 2013). To examine the potential effect of excessive Rac1 activity on NCCs migration, we carried out whole-mount *in situ* hybridization with anti-*Sox10* mRNA probe. Our results revealed reduced *Sox10* expression in the mutant midbrain, hindbrain, and prospective trigeminal ganglia (Figures 6A,B, *n* = 3). To further identify the migrating routes and patterning of neural crest cells *in vivo*, we have performed immunostaining using anti-neurofilament-L antibody, which labels NCCs derived neurofilament (Nie et al., 2018). At E10.5, whole-mount staining results revealed decreased intensity of neurofilament expression in mutant midbrain region (blank arrows in Figures 6C,D, *n* = 7). These results are in line with the whole-mount *in situ* hybridization data, and both support the notion that constitutive activation of Rac1 decreases neural crest cells population at their original destination. In addition, the immunostaining result also showed altered neurofilament expression pattern in mutant pharyngeal arches (green arrows in Figures 6C,D). Sections of these embryos confirmed that in the mutant embryos, the neurofilament-L staining is decreased in the roof of brain (blank arrows in Figures 6G,H) and adjacent tissues (blank arrows in Figures 6I,J), as well as the alteration of its expression pattern in the mutant embryos (green arrows in Figures 6K,L). These results demonstrated that expression of Rac1^*G*12*V*^ alters NCCs migration pattern, and a finely tuned Rac1 activity is required for normal NCCs migration.

### Expression of *Rac1*^*CA*^ Disrupts mDA Progenitor Domain and Neuronal Differentiation in Ventral Midbrain

**FIGURE 7:**
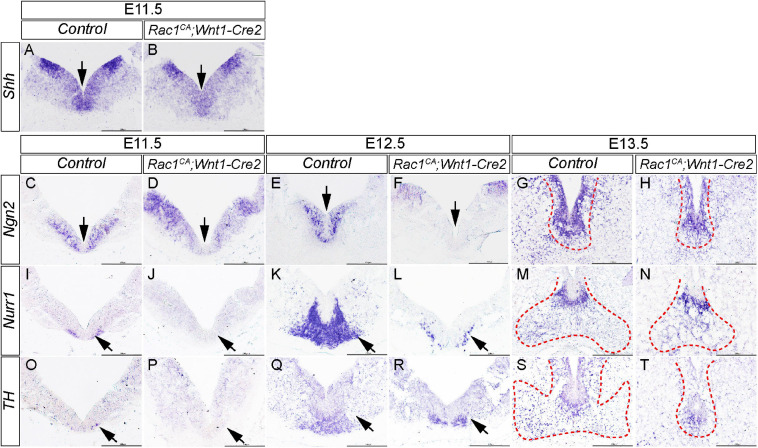
Elevated Rac1 activity disrupts mDA differentiation in ventral midbrain. **(A,B)** *In situ* hybridization with *Shh* antisense probe on coronal sections of E11.5 littermate control and *Rac1^*CA*^;Wnt1-Cre2* embryo. Arrows point to ventral midbrain. **(C–T)** *In situ* hybridization with antisense probes of *Ngn2* **(C–H)**, *Nurr1* **(I–N)**, and *TH* **(O–T)** on coronal sections of littermate control and *Rac1^*CA*^;Wnt1-Cre2* embryos at E11.5, E12.5, and E13.5, respectively. Arrows point to ventral midbrain. Positive signals in **G,H,M,N,S,T** are encompassed with red dashed line. Scale bar in **A–T**, 200 μm.

While Rac1 is known to be essential for mDA differentiation (Čajánek et al., 2013; Hua et al., 2015; Kim et al., 2018), it remains unclear whether or how augmented Rac1 activity affects mDA development. To address this question, we have examined expression of the key markers of mDA differentiation in the ventral midbrain of *Rac1^*CA*^;Wnt1-Cre2* mice and controls. In the control embryos, *in situ* hybridization results show that *Shh* is expressed in the ventral midbrain (arrow in Figure 7A) and lateral midbrain at E11.5. At this stage, expression of *Ngn2*, *Nurr1*, and low level of *TH*, are also detected in the ventral midbrain (Figures 7C,I,O), showing that most mDA are at progenitor stage. Compared to the control, the mutant embryos exhibit decreased expression of *Shh*, *Ngn2*, and *Nurr1* at the ventral midbrain (arrow in Figures 7B,D,J), suggesting fewer mDA progenitors are generated in the mutant embryos. At E12.5, control embryos showed expression of *Ngn2* and *Nurr1* in the ventral midbrain (arrow in Figures 7E,K), and *TH* expression emerges at the same location (arrow in Figure 7Q), demonstrating that some mDA already undergo terminal differentiation at this stage. In the mutant embryos, *Ngn2* expression is barely detected in the ventral midbrain (arrow Figure 7F). Expression of *Nurr1* and *TH* is detected in the mutant, but in a more restricted domain compared to that in the control (arrow in Figures 7L,R). At E13.5, expression of *Ngn2* and *Nurr1* is mostly restricted to the midline in the control embryos, while *TH* expression is detected broadly in the ventral midbrain neurons, indicating most mDA reach terminal differentiation (Figures 7G,M,S). In contrast, in the mutant embryos, expression level of these markers is decreased and their expression becomes restricted to a narrow area at the midline (Figures 7H,N,T). Importantly, *TH* expression in the ventral midbrain, away from the midline progenitor domain, is greatly reduced in mutants compared to controls indicating that only a few TH+ differentiated mDA reach their destination in the mutants. These data suggest augmented Rac1 activity impairs mDA progenitor formation and differentiation *in vivo*. Together our results indicate that a rigorous control of Rac1 activity is required to ensure normal NCCs and mDA development.

## Discussion

Rac1 plays an important role in development and homeostasis. While multiple mutations in *RAC1* allele have been correlated to tumorigenesis, it remains unclear whether elevated Rac1 activity affects embryonic development. In this study, the generation of *Rac1^*CA*^;Wnt1-Cre2* mice enables evaluation of elevated Rac1 activity on NCCs development *in vivo*. Our results show that *Rac1^*CA*^;Wnt1-Cre2* mice exhibit increased proliferation rate, altered cell density, and migration pattern of NCCs. In addition, expression of constitutive active Rac1 also disrupts normal mDA precursor regionalization and neuronal differentiation in the ventral midbrain. These findings demonstrate a rigorous control of Rac1 activity is required for normal NCCs development.

Rac1 regulates multiple cell processes during mouse development, and its regulation seems to be cell context-dependent. *Rac1* null mutant mice die by E9.5, accompanied by ectopic cell death and disrupted germ layers (Sugihara et al., 1998). *Wnt1Cre* mediated NCCs specific *Rac1* depletion causes embryonic lethality at E12.5, and the conditional knockout embryos exhibit severe mid-facial clefting and cardiovascular malformation (Fuchs et al., 2009; Thomas et al., 2010). While both groups reported attenuated cell proliferation rate in the conditional knockout mice NCCs, ectopic cell death was not observed in both studies, indicating that *Rac1* is essential for cell proliferation and normal cell cycle progress of NCCs. In line with these findings, our previous study also revealed chemical inhibition of Rac1 activity represses NCCs proliferation in explant culture (He and Soriano, 2013). In the present study, our results show that expression of Rac1^*G*12*V*^, a constitutive active form of Rac1, increases cell proliferation rate in ventral midbrain (Figures 5A–E). Together these data support the notion that Rac1 is a positive regulator of NCCs proliferation in both loss-of-function and gain-of-function mouse models. Interestingly, although *Rac1* NCCs conditional knockout mice exhibit severe craniofacial and cardiovascular defects, NCCs migration was not affected (Fuchs et al., 2009; Thomas et al., 2010). On the other hand, it is observed that NCCs migration pattern, represented by neurofilament L expression, was altered in *Rac1^*CA*^;Wnt1-Cre2* embryos (Figure 6). These results indicate *Rac1* is not required for NCC migration, but ectopic Rac1 activity disrupts NCCs derivative patterning *in vivo*.

**FIGURE 8:**
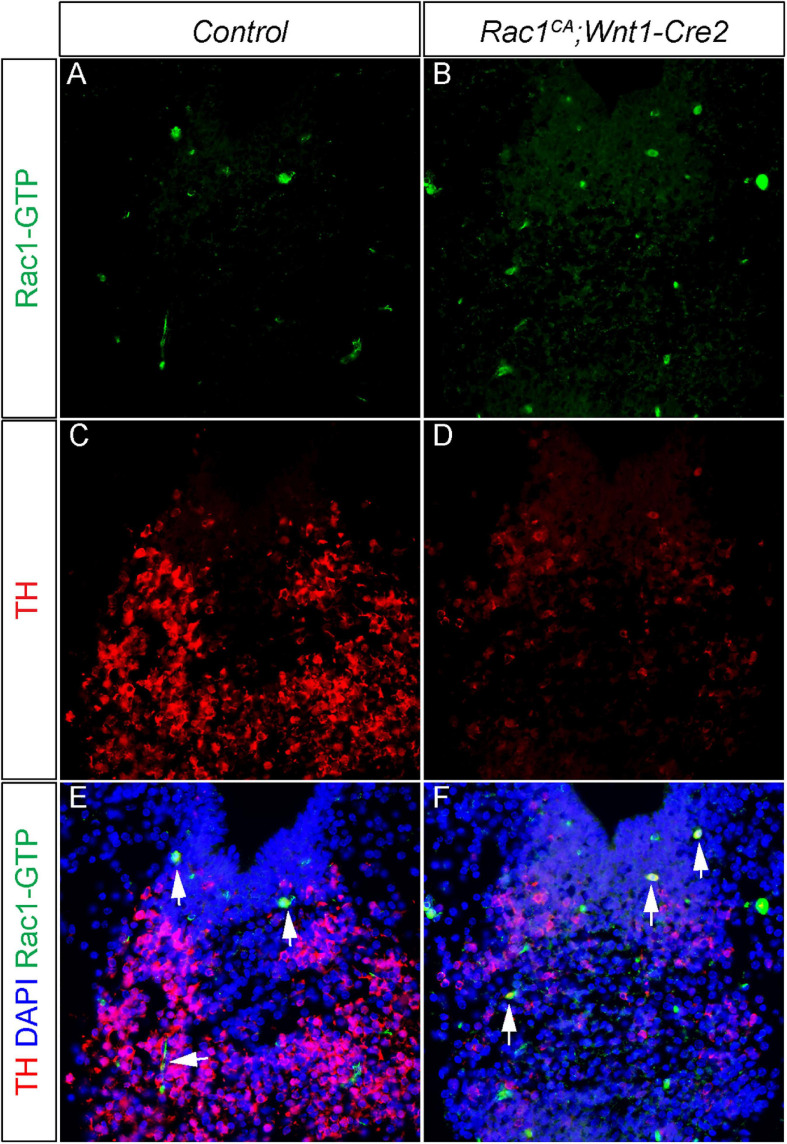
Neural crest driven Rac1^*G*12*V*^ expression regulates mDA differentiation in a non-autonomous manner. **(A,B)** Immunostaining with anti Rac1-GTP antibody (green) on coronal sections of E13.5 littermate control and *Rac1^*CA*^;Wnt1-Cre2* embryo at the midbrain level. **(C,D)** Immunostaining with anti TH antibody (red) on same sections of **A,B**. **(E,F)** Merged images of **A–D** with DAPI staining (blue). Blank arrows point to cells express both TH and Rac1-GTP.

Our results revealed that increased Rac1 activity in NCCs causes disrupted mDA development in the ventral midbrain (Figure 7), suggesting two potential mechanisms of Rac1 regulation in mDA development. In the first scenario, dysregulated Rac1 activity might affect mDA differentiation in an autonomous manner. This model is supported by previous studies showing that Wnt5a engaged Rac1 activity is required to maintain *TH* expression of mDA (Andersson et al., 2008; Čajánek et al., 2013). However, it has not been tested whether increased Rac1 activity affects *TH* expression or mDA differentiation. In the second model, Rac1 activation might lead to extended maintenance of mDA-incompetent domain. This model is supported by multiple lines of evidence. First, we showed increased cell proliferation rate and decreased expression of markers of mDA progenitors in ventral midbrain of *Rac1^*CA*^;Wnt1-Cre2* mutant (Figures 5, 7). These data indicate a change of mDA progenitor domain in the mutant. This is likely because our data show that neurofilament L+ neurons are actually misrouted in the mutant embryos (Figure 6). In addition, the midbrain phenotype of *Rac1^*CA*^;Wnt1-Cre2* is strikingly consistent with that of *Wnt1-Cre^*Tg/Tg*^*, which misexpresses a truncated but functional *Wnt1* mRNA in the midbrain (Lewis et al., 2013). Both models exhibit enlarged midbrain phenotype, accompanied by increased cell proliferation and disrupted mDA differentiation. In addition, *Tiam1*, which encodes a GEF activating Rac1 specifically, is an established Wnt responsive gene (Malliri et al., 2006). It is thus likely that a Wnt1/Tiam1/Rac1 axis promotes cell proliferation rate and expansion of the mDA-incompetent domain and disrupted normal midbrain development and mDA differentiation. It would be interesting to test whether *Tiam1* expression and Rac1 activity increases in *Wnt1-Cre^*Tg/Tg*^* ventral midbrain, and whether Rac1-GTP and TH are co-localized in the differentiating mDA. To this end, we have carried out double immunostaining using anti-TH antibody and anti-Rac1-GTP antibody in the ventral midbrain of the control and mutant embryos. We found the mutant ventral midbrain exhibit increased expression of Rac1-GTP and dramatic decrease of TH expression (Figures 8A–F). However, there is only few cells co-incidentally express both proteins. This result support the notion that neural crest expressed Rac1^*G*12*V*^ regulates mDA differentiation in a non-autonomous manner.

In summary, we presented an *in vivo* model to study augmented Rac1 activity in midbrain morphogenesis and mDA development, and our data show that elevated Rac1 activity alters NCCs proliferation and migration pattern, as well as mDA differentiation in the ventral midbrain. These findings shed light on the mechanisms underlying brain tumors correlated with mutations in *RAC1* allele.

## Data Availability Statement

The raw data supporting the conclusions of this article will be made available by the authors, without undue reservation.

## Ethics Statement

The animal study was reviewed and approved by the Institutional Animal Care and Use Committee of Tulane University.

## Author Contributions

AG and FH: conceptualization, methodology, and writing. AG, CD, GB, and MG: experiment and data analysis. All authors contributed to review and approved the submission.

## Conflict of Interest

The authors declare that the research was conducted in the absence of any commercial or financial relationships that could be construed as a potential conflict of interest.

## Publisher’s Note

All claims expressed in this article are solely those of the authors and do not necessarily represent those of their affiliated organizations, or those of the publisher, the editors and the reviewers. Any product that may be evaluated in this article, or claim that may be made by its manufacturer, is not guaranteed or endorsed by the publisher.
